# NHE1-Mediated Metabolic Reprogramming in Cancer

**DOI:** 10.3390/metabo16030195

**Published:** 2026-03-15

**Authors:** Majd A. Al-Hamaly, Beau R. Forester, Jessica S. Blackburn

**Affiliations:** 1 Pharmacology and Nutritional Sciences, University of Kentucky, Lexington, KY 40356, USA; m.hamaly@uky.edu; 2 Markey Cancer Center, University of Kentucky, Lexington, KY 40536, USA; beau.forester@uky.edu; 3 Molecular and Cellular Biochemistry, University of Kentucky, Lexington, KY 40356, USA

**Keywords:** NHE1, cancer metabolism, ion exchangers, pH regulation, mitochondria, OXPHOS

## Abstract

The sodium–hydrogen exchanger-1 (NHE1) is a ubiquitously expressed transmembrane transporter that plays a central role in maintaining intracellular pH homeostasis and supporting normal cellular function. In cancer, NHE1 is overexpressed in many tumor types and has been associated with increased cancer cell metastasis and proliferation. Beyond these established roles, emerging evidence implicates NHE1 as a regulator of cancer cell metabolism. By driving intracellular alkalinization and shaping the tumor microenvironment, NHE1 influences metabolic pathway activity, mitochondrial function, redox balance, and cellular stress responses. In this review, we synthesize current evidence linking NHE1 dysregulation to metabolic reprogramming in cancer, with a focus on mitochondrial metabolism, glycolytic flux, lysosomal biology, and reactive oxygen species-associated stress pathways. We further evaluate pharmacological strategies targeting NHE1, emphasizing their metabolic consequences, translational potential, and the challenges that have limited clinical application to date. Collectively, this review highlights NHE1 as a potential integrator of ion transport and metabolic control in cancer and discusses how targeting NHE1-driven metabolic programs may support the development of novel therapeutic strategies.

## 1. Introduction

The sodium–hydrogen exchanger (NHE) family of proteins is ubiquitously expressed in the plasma membrane of eukaryotic cells and is responsible for maintaining intracellular pH balance through the exchange of intracellular H^+^ for extracellular Na^+^ [1]. Nine NHE isoforms have been identified (NHE1–NHE9), with each subtype exhibiting distinct tissue distribution and functional characteristics. While NHE2–NHE9 are predominantly tissue-specific in organs such as the kidney, small intestine, and the brain, NHE1 is expressed in most tissue types and remains the most widely studied isoform [2]. In addition to its central role in pH regulation, NHE1 contributes to diverse cellular processes, including migration, proliferation, adhesion, and apoptotic signaling [3]. Notably, elevated NHE1 expression has been reported across multiple cancer types compared to normal tissue and is frequently associated with increased tumor aggressiveness [4]. This association is consistent with the inverted pH gradient observed in many tumors, characterized by alkaline intracellular and acidic extracellular environments, in which NHE1 plays a major role by promoting proton extrusion [5].

Evidence from patient-derived tumor samples supports a strong clinical association between NHE1 expression and cancer progression. In ovarian cancer, immunohistochemical analyses revealed significantly higher NHE1 expression compared with benign lesions and normal controls [6]. Similarly, in brain tumors, elevated mRNA levels of *SLC9A1*, the gene encoding NHE1, were associated with higher glioma grade and shorter overall survival [7]. Consistent correlations between elevated NHE1 protein or gene expression and worse clinical outcomes have also been described in esophageal [8], gastric [9], and breast cancers [10], underscoring the critical contribution of this ion exchanger to tumorigenesis and disease progression.

Although the exact mechanisms by which NHE1 promotes oncogenic phenotypes are not fully understood, emerging evidence supports a strong link between NHE1 dysregulation and metabolic adaptation in cancer cells. Many metabolic enzymes are pH-sensitive, and NHE1-driven intracellular alkalinization can directly shift flux through major biosynthetic and energy pathways [11,12,13,14], triggering a cascade of downstream metabolic consequences [15]. These changes include enhanced carbon anabolism and biosynthetic flux [16], shifts in energy metabolism between oxidative phosphorylation (OXPHOS) and glycolysis [17], and disturbed mitochondrial function and homeostasis [18]. In addition to the pH-dependent metabolic consequences of NHE1 perturbation, emerging evidence supports pH-independent functions of NHE1 that influence cellular energy regulation [19]. Further, NHE1 can act as a structural scaffold, physically and functionally interacting with metabolic enzymes to modulate cellular metabolism [10]. Collectively, these metabolic reprogramming events can profoundly influence tumor behavior and response to therapy. Consistent with this, metabolic plasticity allows cancer cells to adapt to conditions of nutritional stress, evade apoptosis, and survive chemotherapy treatment [20]. Treatment-resistant cells frequently exhibit energetic and biosynthetic metabolic shifts, highlighting the importance of metabolic reprogramming in cancer stress responses [21].

Despite these insights, the precise interplay between ion exchangers, cancer metabolism, and disease progression remains incompletely understood. In this review, we synthesize emerging evidence linking NHE1 to metabolic reprogramming in cancer, an area that has not been comprehensively integrated despite recent advances. A deeper understanding of how NHE1 and related ion exchangers shape the cancer metabolic landscape has the potential to reveal new therapeutic vulnerabilities and provide broader insights into tumor adaptation and treatment resistance.

## 2. NHE1 Is Implicated in Tumor Development and Progression

Beyond its canonical role as a plasma membrane ion exchanger, NHE1 has emerged as a multifunctional regulator of cancer cell behavior. This role is supported by elevated NHE1 expression and activity observed across diverse tumor types in patient samples [6,7,8,9,10]. Experimental evidence from multiple model systems further demonstrates a strong association between NHE1 and key cancer-associated processes, including oncogenic transformation, sustained proliferation [22], therapeutic drug resistance [23], migration and invasion [9] and remodeling of the tumor microenvironment [24]. Importantly, genetic or pharmacological perturbation of NHE1 consistently attenuates these tumor-promoting phenotypes in vitro and in vivo (Table 1), positioning NHE1 as an active driver of cancer progression rather than a passive marker. This section synthesizes evidence from diverse model systems to highlight the cancer-associated processes through which NHE1 contributes to tumor development and progression across solid and hematologic malignancies.

### 2.1. Oncogenic Transformation and Proliferation

Early studies established NHE1 as a direct contributor to oncogenic transformation and increased proliferative capacity in cancer cells. Reshkin et al. used the E7 oncogene to induce malignant transformation in NIH3T3 fibroblasts and HPKIA human keratinocytes, and demonstrated that cytoplasmic alkalinization, a known permissive factor for cell cycle progression, was a defining feature of the transformation process [34]. Specifically, activation of E7 using a tetracycline-controlled system resulted in increased intracellular pH, which was shown to be NHE1-dependent, as it was blocked by two NHE1 inhibitors and was not affected by inhibition of the bicarbonate transporter. These findings suggest that while E7 activation precedes NHE1 activation and subsequent alkalinization, NHE1-mediated pH elevation is necessary to drive the transformation phenotypes in this model. Importantly, treatment of nude mice with NHE1 inhibitors significantly delayed tumor development in vivo [34].

Subsequent studies extended these observations to additional malignancies. In gastric cancer, Liu et al. demonstrated that treatment of SGC-7901 cells with an antisense NHE1 vector reduced proliferative capacity, increased apoptosis, and suppressed tumor formation in nude mice [35]. These findings were later confirmed by Xie et al., who employed both genetic shRNA knockdown of NHE1 and pharmacological inhibition with ethyl-N-isopropylamiloride (EIPA) [9]. Treatment with EIPA effectively reduced the intracellular steady state pH and the pH recovery rate. NHE1 inhibition suppressed cellular proliferation by disrupting cell cycle progression, accompanied by reduced expression of key cell cycle regulators, including Cyclin D1 and Cyclin B1. While intracellular pH coincided with these changes, the study did not directly establish whether cyclin regulation was mediated by pH-dependent mechanisms or secondary signaling pathways. Importantly, EIPA treatment significantly reduced gastric cancer growth in vivo compared with controls [9].

The role of NHE1 in sustaining proliferation is not restricted to solid tumors. In acute myeloid leukemia (AML), primary patient samples and cytarabine-resistant cell lines exhibited elevated intracellular pH relative to normal bone marrow mononuclear cells. This alkalinization was correlated with elevated NHE1 expression. Treatment with the NHE1 inhibitor hydroxylmethyl amiloride (HMA) blocked cellular proliferation and induced apoptosis, even in therapy-resistant cells, potentially through effects on MAPK signaling pathways [36]. Similarly, Jiang et al. reported that inhibition of NHE1 activity by HMA suppressed growth across multiple AML lines through cell cycle modulation [30].

Importantly, while the majority of the NHE1 cancer literature supports a tumor-promoting function of NHE1, work in esophageal squamous cell carcinoma (ESCC) suggests a tumor-suppressive function in certain contexts. In TE2 and TE5 ESCC cell lines, NHE1 knockdown promoted proliferation and decreased apoptosis, while overexpression had the opposite effects [8]. These findings contrast with reports in most other tumor types and suggest that the functional consequences of NHE1 activity may be context dependent and potentially influenced by the tumor-specific molecular characteristics or differences in pH regulatory networks.

### 2.2. Migration, Invasion, and Epithelial–Mesenchymal Transition (EMT)

NHE1 has been implicated in regulating cancer cell migration and invasion, which are key processes underlying metastatic dissemination. In gastric cancer, Xie et al. reported that NHE1 inhibition reduced cellular migration and invasion, accompanied by altered expression of epithelial–mesenchymal transition markers, including E-cadherin and vimentin [9]. In breast cancer, Amith et al. demonstrated that dysregulated NHE1 activity promotes metastatic behavior and that NHE1 inhibition reduces both cell viability and invasiveness [37]. Consistent with this, knockout of NHE1 in MDA-MB-231 breast cancer cells resulted in significantly smaller tumors and in reduced engraftment compared with wild-type controls [38].

In pancreatic cancer, Cardone et al. showed that Epidermal Growth Factor (EGF) stimulation induced NHE1-dependent phenotypes, including colony formation ability, invasion, and motility across multiple pancreatic cancer cell lines [28]. These investigations were supported by an analysis of patient datasets identifying a strong correlation between EGFR signaling and *SLC9A1* expression. Notably, the researchers identified the formation of a dynamic complex between EGFR, NHE1, and the scaffolding protein Na^+^/H^+^ exchanger regulatory factor 1 (NHERF1). This complex plays a central role in differentiating NHE1 function across different regions of the plasma membrane to promote invasion and 3D tumor formation [28]. Complementary work by Wang et al. demonstrated that AKT-mediated upregulation of LAMC2 (Laminin Subunit Gamma 2) promoted NHE1 expression, activity and membrane accumulation, leading to increased extracellular acidification and actin-dependent pseudopodia formation, phenotypes associated with enhanced pancreatic cancer cell migration and invasion [24].

In T-cell acute lymphoblastic leukemia (T-ALL), Altaf et al. described an ezrin-mediated mechanism through which NHE1 promotes cellular polarization, invasion, and therapy resistance [31]. Inhibition of either NHE1 or ezrin reduced cellular polarization, while pharmacological inhibition of NHE1 with cariporide significantly decreased chemotaxis of MOLT-4 cells, highlighting the role of NHE1 in leukemic cell migration [31]. Notably, the colocalization of NHE1 and ezrin is driven by stimulation with the chemokine CCL25. While NHE1 functions as an anchor for actin filaments, contributing to adhesion and cytoskeletal organization, its interaction with ezrin appears to serve a distinct role. Specifically, the NHE1-ezrin complex functions as a signaling scaffold that facilitates signal propagation, distinguishing it from the more structural NHE1-actin interaction [31,39,40]. These findings underscore the dynamic interplay between NHE1 activity, cytoskeletal organization, growth factor signaling, and microenvironmental remodeling during tumor invasion.

Collectively, these studies indicate that NHE1-driven migration and invasion are mediated through multiple, context-dependent mechanisms. Intracellular alkalinization may promote cytoskeletal remodeling and cell cycle progression, while extracellular acidification can facilitate extracellular matrix degradation and microenvironmental remodeling. In parallel, NHE1 also functions as a signaling scaffold, forming dynamic complexes with proteins such as EGFR, NHERF1, and ezrin to coordinate pro-invasive signaling pathways. Thus, invasive phenotypes likely reflect integrated transport-dependent and transport-independent functions of NHE1 rather than a single dominant mechanism.

### 2.3. Drug Resistance and Therapy Sensitization

An additional recurring theme across cancer types is the contribution of NHE1 to therapeutic resistance and treatment response. In breast cancer models, inhibition of NHE1 potentiated chemotherapeutic efficacy [37]. Combination treatment with paclitaxel and low-dose HMA resulted in significantly greater cell death compared with paclitaxel or HMA alone [38]. Similar sensitization effects were observed in pancreatic cancer, where the NHE1 inhibitor cariporide synergized with the EGFR inhibitor erlotinib to suppress three-dimensional growth and invadopodia formation. The combination achieved a dose reduction index of 2.6-5.2, depending on EGF stimulation [28].

In AML, combination therapy with venetoclax and HMA had a pronounced anti-leukemic effect in mouse xenograft models and primary patient samples. This synergy was linked to lysosomal involvement, supported by gene-enrichment analyses and increased expression of the lysosomal marker LAMP1. Transmission electron microscopy further revealed increased lysosome number and volume in the combination-treated cells compared to the controls [30]. A similar lysosome-mediated mechanism of NHE1-dependent cell death was described in multiple myeloma (MM), where NHE1 expression strongly correlated with disease aggressiveness and shorter patient survival. In MM cell lines, Yang et al. demonstrated that HMA treatment increased the number of lysosomes as measured by LysoTracker staining and electron microscopy, along with increased expression of LAMP1 [33]. Further, HMA treatment inhibited proliferation, induced apoptosis, and sensitized cells to carfilzomib therapy in a dose-dependent manner. These effects were associated with increased lysosomal biogenesis and permeability, providing a potential mechanism for enhanced apoptosis [33]. Mechanistically, both studies found that HMA- or combination-treatment-induced cell death occurred through a TFE3-mediated lysosomal mechanism [30,33]. While it is possible that the lysosomal phenotypes described in the AML study were amplified by combination therapy, the observation of consistent lysosomal alterations in MM following single-agent HMA treatment suggests that these effects are at least partially attributable to NHE1 inhibition rather than solely to a generalized stress response. However, since the findings in the AML study were primarily derived from combination therapy, and given the lack of genetic inhibition experiments in both the AML and MM studies, it remains difficult to establish a direct causal link between NHE1 activity and lysosomal alterations.

**Figure 1:**
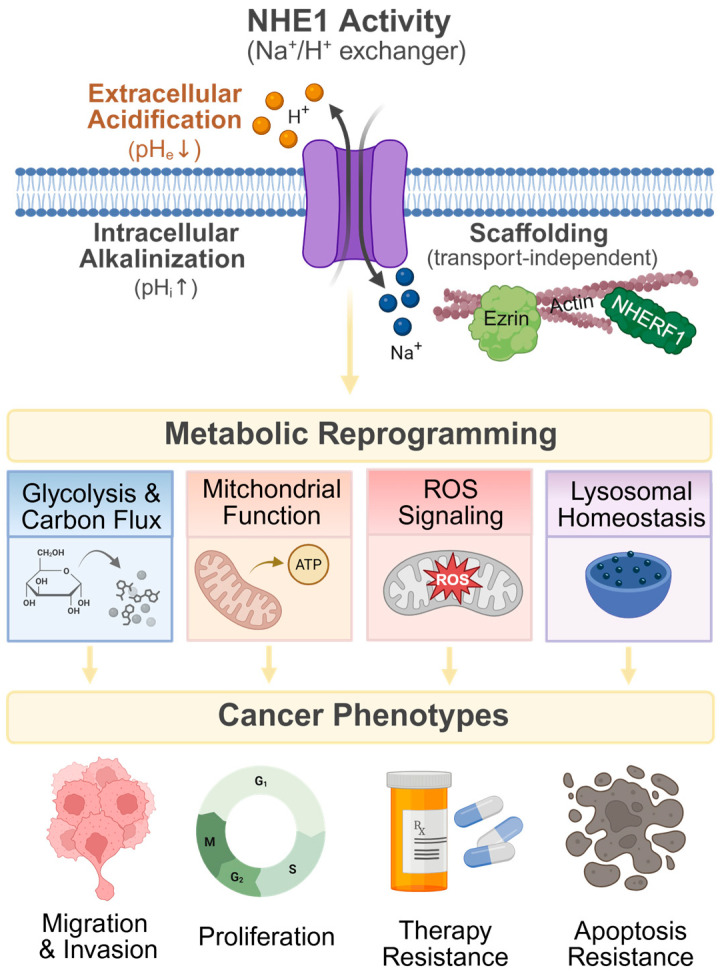
NHE1 regulates diverse metabolic alterations and cancer-associated phenotypes through its ion exchange and scaffolding functions. NHE1-mediated downstream effects occur primarily through its ion transport activity and proton extrusion (Na^+^ influx and H^+^ efflux), which promote intracellular alkalinization and contribute to tumor microenvironment acidification. An emerging role of NHE1 involves its scaffolding function and physical interactions with proteins such as actin, ezrin, and Na^+^/H^+^ exchanger regulatory factor 1 (NHERF1). These activities are associated with alterations in carbon flux, redox balance (ROS), mitochondrial function, and lysosomal homeostasis. Together, these metabolic adaptations contribute to the emergence and maintenance of multiple cancer phenotypes across solid tumors and hematologic malignancies.

Taken together, data from both solid and hematologic malignancies highlight a broad and conserved role for NHE1 in tumorigenicity, progression, invasion, and therapy resistance (Table 1 and Figure 1). However, the mechanistic basis through which NHE1 drives these diverse phenotypes remains incompletely understood. Given the central role of metabolic rewiring in cancer, metabolic adaptation may represent a unifying mechanism underlying the widespread impact of NHE1 across tumor types. In the following section, we synthesize current evidence describing the metabolic consequences of NHE1 dysregulation.

## 3. NHE1 Is a Contributor to Cancer Metabolism

Cancer cells exhibit altered metabolic activities compared with normal cells. These changes enhance cellular fitness and confer selective advantages that support proliferation, survival, and adaptation to environmental stress. These metabolic alterations enable cancer cells to meet increased energetic and biosynthetic demands and are now recognized as a core hallmark of cancer [41,42]. Extensive efforts have sought to therapeutically exploit the metabolic differences between normal and malignant cells, leading to the development of several approved metabolic therapies and numerous additional agents currently in clinical or preclinical development [43]. These advances underscore the importance of continued investigation into the regulatory mechanisms that coordinate cancer cell metabolism.

Intracellular pH is a critical determinant of metabolic behavior and is frequently dysregulated in cancer cells relative to untransformed counterparts [13]. The inverted pH gradient characteristic of many tumors, characterized by elevated intracellular pH coupled with extracellular acidification, has been implicated in early tumorigenesis and disease progression [44]. Beyond these cancer-associated phenotypes, intracellular pH directly influences metabolic enzyme activity, nutrient transport, mitochondrial function, and global metabolic flux [45].

NHE1 is among the most extensively studied regulators of intracellular pH [46]. Beyond its canonical role in proton extrusion, NHE1 activity shapes the tumor microenvironment [47], modulates intracellular signaling pathways [48,49], and influences organelle biology [39]. Through these interconnected mechanisms, NHE1 functions as an integrator of metabolic programs that support cancer cell bioenergetics, carbon anabolism, and adaptive stress responses (Figure 1) [39,50]. In the following subsections, we synthesize the current evidence linking NHE1 activity to key metabolic alterations, with a focus on mitochondrial remodeling, carbon anabolism, lysosomal biology, and stress response pathways.

### 3.1. NHE1 Regulation of Mitochondrial Function, Lysosomal Homeostasis and Stress Responses

Across multiple tumor types, perturbation of NHE1 activity consistently disrupts mitochondrial structure, energy production, and redox balance, implicating NHE1 as a potential contributor to mitochondrial homeostasis. In breast cancer cells, treatment with the NHE1 inhibitor HMA induced mitochondrial swelling and fragmentation, as evidenced by electron microscopy. Notably, co-treatment with lysosomal protease inhibitors or reactive oxygen species (ROS) scavengers rescued cell viability following HMA exposure, suggesting functional coupling between mitochondrial integrity, lysosomal function, and oxidative stress response. Mechanistically, the authors demonstrated that HMA treatment induced JNK phosphorylation while reducing AKT and ERK phosphorylation. Importantly, these effects were reversed by the ROS inhibitor N-acetylcysteine, indicating that this pro-death signaling cascade is downstream of ROS [51]. Importantly, the attenuation of cellular cytotoxicity upon treatment with lysosomal protease inhibitors supports a functional contribution of lysosomes to cell death, rather than a purely protective mechanism against cellular stress. It is also possible that the lysosomal contribution shifts from a stress-protective to a pro-death response upon reaching a critical threshold of stress or mitochondrial damage. Clarifying the precise role of lysosomes is an area that requires further mechanistic investigation.

Similar observations have been reported in pancreatic cancer models, where NHE1 inhibition disrupted mitochondrial respiration without altering core metabolic pathway utilization. Treatment with EIPA reduced oxidative phosphorylation relative to tissue-matched untransformed cells, while glycolytic flux remained unchanged, as evidenced by stable lactate secretion and intracellular pyruvate levels. The inhibition of NHE1 by EIPA was confirmed by the loss of intracellular pH recovery following an acid load, indicating the substantial contribution of NHE1 to the pH at steady state in those cells. Further investigation of mitochondrial fuel usage showed that EIPA treatment did not significantly alter the relative contributions of fatty acid oxidation, glutaminolysis, or glycolysis to basal oxygen consumption, nor did it affect mitochondrial membrane potential. Instead, NHE1 inhibition with EIPA was associated with pronounced changes in mitochondrial morphology, characterized by increased fusion and the formation of fewer, larger mitochondria with expanded tubular networks [52]. Together, these findings suggest that NHE1 inhibition primarily disrupts mitochondrial structural organization rather than substrate utilization, uncoupling mitochondrial morphology from canonical metabolic flux.

Additional evidence linking NHE1 to mitochondrial metabolism and oxidative stress has emerged from studies in colon cancer, where NHE1 inhibition altered mitochondrial subcellular localization. HMA treatment induced redistribution of mitochondria toward the nuclear membrane, rather than being evenly distributed throughout the cytoplasm. This was accompanied by a time-dependent increase in ROS production and progressive mitochondrial membrane depolarization. Treatment with antioxidants was effective in reversing the HMA-triggered production of ROS [18]. Consistent findings have been reported in mesothelioma, where cariporide treatment was associated with an increase in ROS levels and in the proportion of cells exhibiting loss of mitochondrial membrane potential [53]. In glioma cells, treatment with the amiloride derivative UCD38B (5′−benzylglycinyl-amiloride) likewise decreased mitochondrial membrane potential [54].

In addition to cancer cell-intrinsic effects, NHE1 also regulates metabolic programs within immune cells of the tumor microenvironment. In glioma-associated microglia (GAM), pharmacological inhibition of NHE1 modulated immunosuppressive phenotypes. Combination treatment of GAM with cariporide and temozolomide (TMZ) resulted in a two-fold reduction in extracellular acidification rate (ECAR) and a two-fold increase in oxygen consumption rate (OCR), indicative of a metabolic shift away from glycolysis toward oxidative phosphorylation. This metabolic reprogramming was accompanied by an increase in mitochondrial mass and enhanced glucose uptake (2-NBDG), suggesting greater reliance on mitochondrial metabolism rather than increased glycolytic flux. To test the functional relevance of these metabolic changes in vivo, NHE1 was conditionally deleted in Cx3cr1^+^ myeloid cells. Following TMZ treatment, NHE1-deficient mice exhibited significantly reduced myeloid cell infiltration and enhanced PD-L1 expression compared to controls. Furthermore, combination treatment with TMZ and anti-PD-1 significantly prolonged survival in NHE1 knockout mice, indicating that NHE1-dependent metabolic reprogramming of myeloid cells may contribute to immunosuppression in glioma [17]. These findings support a cell-intrinsic role for NHE1 in regulating immune cell metabolism, although NHE1-driven extracellular acidification within the tumor microenvironment may also indirectly influence immune cell behavior.

Collectively, these studies support a broad role of NHE1 in regulating mitochondrial function, redox balance, and stress responses in both cancer cells and components of the tumor microenvironment. Across multiple tumor types, NHE1 influences mitochondrial energy production, localization, membrane potential, and structural integrity (Table 2). These mitochondrial effects can be explained, at least in part, by the impact of NHE1 on intracellular pH, given the strong dependence of mitochondrial enzyme activity and membrane potential maintenance on tightly regulated pH [55]. Disruption of these processes is frequently accompanied by elevated reactive oxygen species (ROS) production and downstream consequences such as DNA damage [18,53], linking NHE1-dependent pH regulation to oxidative stress and genomic instability.

Importantly, NHE1 involvement in mitochondrial regulation is further supported by studies in non-malignant tissues, particularly in endocrine and cardiac systems, where NHE1 has been extensively investigated [56,57,58]. Notably, NHE1 has been detected on the mitochondrial membrane in cardiomyocytes [59]. However, to date, similar mitochondrial localization of NHE1 has not been reported in cancer cells. Determining whether NHE1 localizes to mitochondria in malignant contexts is a critical, underexplored area of investigation that could provide mechanistic insight into the consistent mitochondrial phenotypes observed following NHE1 inhibition. Establishing this link would be important for refining our understanding of NHE1 as a therapeutic target in cancer and could inform more rational combination strategies with metabolic- or mitochondrial-targeting agents.

### 3.2. NHE1 in Modulating Carbon Metabolism

In addition to regulating mitochondrial energy metabolism, NHE1 also influences cellular carbon metabolism. In AML cells, inhibition of NHE1 using HMA induced intracellular acidification and reduced glucose uptake. These metabolic effects were linked to impaired proliferation, as supplementation with ribose and pyruvate rescued the antiproliferative effects of NHE1 inhibition. Conversely, NHE1 overexpression increased glucose uptake and proliferation in normal hematopoietic stem and progenitor cells. NHE1-mediated intracellular alkalinization promotes carbon utilization pathways that support cell growth and proliferation. This was also observed upon the overexpression of another proton exporter, Monocarboxylate transporter 4 (MCT4). Collectively, these findings demonstrate that changes in intracellular pH can reprogram critical enzymes to favor carbon anabolism [16].

Importantly, the expression of the glycolytic enzyme hexokinase 2 (HK2) is correlated with high NHE1 activity in murine T helper cells [60]. Moreover, Singh et al. identified elevated intracellular pH and increased NHE1 expression in Th9 cells compared to other T-cell subsets [61]. Th9 cells are known to exert protective anti-tumor effects and are characterized by their production of IL-9. Researchers also observed elevated expression of key glycolytic genes, including glucose transporter 1 (Glut1) and HK2, supporting an increased glycolytic rate in Th9 cells relative to other immune subsets. Functionally, knockdown of NHE1 in Th9 cells using siRNA decreased intracellular pH, impaired ATP production, and reduced IL-9 expression. Inhibition of AKT decreased NHE1 activity and IL-9 production, suggesting a role for NHE1 upregulation in supporting Th9 metabolic and functional activity [61].

These findings highlight a metabolism-centered role for NHE1, likely mediated through its regulation of intracellular pH, that influences tumor microenvironment immunity. Together with the work by Hasan et al. on GAMs [17], these data suggest that NHE1-mediated metabolic regulation extends beyond tumor cells to influence immune cell function and therapeutic response within the tumor microenvironment. This emerging evidence supports the potential for targeting NHE1 to enhance the efficacy of immune-based therapies.

### 3.3. pH-Independent Roles of NHE1 in Metabolic Regulation

The impact of NHE1 on cellular metabolism may not be explained solely by changes in steady-state intracellular pH but may instead reflect a requirement for NHE1 activity during metabolic recovery from stress. Early work by Glunde et al. investigated the effects of NHE1 inhibition under conditions of extracellular acidosis using rat glial cell lines [19]. Extracellular acidification induced a reversible suppression of cellular energy metabolism, as evidenced by reduced phosphocreatine-to-creatine (PCr/Cr) ratios and decreased levels of glycolytic and TCA cycle metabolites, which recovered upon restoration of physiological pH [19]. In contrast, when NHE1 was inhibited using HOE642 (cariporide), the acidosis-induced reduction in PCr/Cr ratios was exacerbated, and phosphocreatine levels failed to recover despite normalization of intracellular pH. These findings indicate that restoration of intracellular pH alone is not sufficient to fully restore cellular energy metabolism following acid stress, supporting a pH-independent requirement for NHE1 activity in metabolic recovery [19].

Consistent with this model, NHE1 may influence metabolic regulation through mechanisms that extend beyond its canonical ion transport activity. In particular, NHE1 has been reported to physically associate with key metabolic enzymes, suggesting a potential scaffolding role linking ion homeostasis to metabolic regulation. Affinity chromatography coupled with mass spectrometry identified multiple NHE1-interacting partners in triple-negative breast cancer cells. The identified enzymes included glutamate dehydrogenase, fructose-bisphosphate aldolase, transketolase, glyceraldehyde-3-phosphate dehydrogenase, and α-enolase. Importantly, the α-enolase and NHE1 complex was confirmed by co-immunoprecipitation, and was enhanced under serum starvation, a condition in which NHE1 activity is enhanced [10]. Together, these findings support a model in which NHE1 directly participates in metabolic regulation through physical and functional interactions with biosynthetic machinery. These interactions suggest that NHE1 may act as a scaffold linking ion homeostasis to metabolic enzyme activity, extending its role beyond intracellular pH control.

Despite increasing evidence linking NHE1 to metabolic reprogramming in cancer, several limitations of the current literature should be acknowledged. Many studies rely on pharmacological inhibitors of NHE1, including amiloride derivatives, which can exhibit off-target effects and limited isoform specificity. Accordingly, many reported metabolic alterations, including changes in OXPHOS, ROS accumulation and shifts in glycolytic flux, are primarily derived from associative studies rather than direct mechanistic interrogation of NHE1 metabolic function. Complementary approaches such as genetic inhibition studies and rescue experiments to selectively perturb NHE1 activity in metabolic contexts are relatively underutilized. Few studies employ transport-inactive NHE1 mutations or domain-specific disruption strategies that would allow definitive separation of exchanger activity from scaffolding or signaling functions. In the absence of these approaches, it remains challenging to establish direct causality to NHE1 or exclude secondary effects resulting from global changes in intracellular pH.

Nevertheless, the remarkable consistency of metabolic phenotypes observed across diverse cancer types, experimental systems, and inhibitor strategies supports a functional role for NHE1 in regulating cancer metabolism. These limitations highlight a critical need for more selective genetic and pharmacological tools to definitively dissect NHE1-dependent metabolic mechanisms and to enable rational therapeutic targeting.

## 4. Pharmacological Inhibitors of NHE1

Given the emerging role of NHE1 in cancer cell metabolism and therapeutic response discussed above, pharmacological inhibition of NHE1 has been widely explored as both a mechanistic tool and a potential therapeutic strategy in oncology. Various inhibitors of NHE1 have been developed over the past several decades with diverse chemical structures, potencies, and degrees of isoform selectivity [62]. Initially, NHE1 inhibitors were developed and optimized for the treatment of cardiovascular and inflammatory conditions; only later were these agents adopted as experimental tools in cancer research [63]. Below, we summarize the major classes of NHE1 inhibitors, their pharmacological properties, and their translational relevance, with particular emphasis on considerations unique to oncology applications.

### 4.1. Amiloride and Pyrazine Derivatives

#### 4.1.1. Amiloride

Amiloride and its derivatives have been the most widely used pharmacological tools to interrogate NHE1 function in cancer and cancer metabolism models. Amiloride was the first compound identified to inhibit the NHE1 isoform [64]. NHE1 is composed of 815 amino acids, with regulatory N- and C-terminal cytosolic domains and a transmembrane region forming twelve α-helices [65]. Amiloride inhibits NHE1 by binding near Glu346 within the extracellular domain, thereby interfering with Na^+^/H^+^ ion exchange and blocking proton extrusion [66]. However, amiloride displays limited specificity, inhibiting additional NHE isoforms with varying sensitivities, with isoforms 1 and 2 being the most sensitive, isoforms 3 and 4 the most resistant, and isoform 5 showing intermediate inhibition. In addition to its ability to inhibit NHE1, amiloride also inhibits epithelial Na^+^ channels (ENaCs) in the distal nephron [67], the Na^+^/Ca^2+^ exchanger [62], and urokinase-type plasminogen activator (uPA) [68], which is implicated in cancer cell migration, invasion, and metastasis [69]. These off-target activities complicate the interpretation of amiloride-based studies in cancer models and underscore an important limitation of much of the early NHE1 literature, which relied on amiloride as one of the few available pharmacological tools to probe NHE1-dependent tumor phenotypes.

Amiloride is approved by the U.S. Food and Drug Administration (FDA) for the treatment of hypertension and congestive heart failure. It is widely used as a potassium-sparing diuretic that is weakly metabolized by the liver and excreted unchanged in the urine [70]. The clinical effects of amiloride are mediated through its inhibition of epithelial sodium channels (ENaCs) in the kidney. Amiloride has an oral bioavailability of approximately 50%, with peak plasma concentrations achieved 2–3 h after administration and a half-life of 6–9 h [71]. The recommended daily dose of amiloride for approved indications, typically in combination with other diuretics, is 5–10 mg/day and should not exceed 20 mg/day [72].

#### 4.1.2. Pyrazine Derivatives (EIPA, HMA, DMA)

To improve upon the limited specificity and selectivity of amiloride, several pyrazine derivatives were generated through substitutions at the nitrogen of the 5-amino group, yielding 5-(N-ethyl-N-isopropyl)amiloride (EIPA), 5-(N,N-hexamethylene)amiloride (HMA), and 5-(N,N-dimethyl)amiloride (DMA) [62,66]. These modifications substantially reduced inhibitory activity toward ENaCs and the Na^+^/Ca^2+^ exchanger while increasing potency toward NHE1, although isoform selectivity remained incomplete [62].

Despite improved target engagement, both EIPA and HMA have notable in vivo limitations. HMA exhibits a very short apparent half-life of approximately 36 min in mice, consistent with findings reported in rats, rabbits, and pigs. In the same animals, HMA displayed a low oral bioavailability of 4.5%, compared to 47% for amiloride [73]. Similarly, EIPA has a plasma half-life of approximately 31 min following intraperitoneal administration in mice, with rapid hepatic clearance but detectable retention in the kidney and transplanted tumor tissue for up to 2 h [74]. As a result, these derivatives have been widely adopted in cancer and metabolism studies, while their pharmacokinetic properties have limited their advancement beyond experimental systems.

### 4.2. New Experimental NHE1 Inhibitors

Additional NHE1 inhibitors that are structurally independent of amiloride have been developed with improved potency and selectivity. Among the first non-guanidine inhibitors was SL-591227, which exhibits a 30-fold increase in potency compared to cariporide [75]. Phenoxazine derivatives, including Phx-1 and Phx-3, have also been described. These compounds induce a rapid and dose-dependent decrease in intracellular pH across ten different cancer cell lines and display pre-apoptotic and cytotoxic effects following prolonged incubation [76]. More recently, Atwal and colleagues at Bristol-Myers Squibb developed compound 9t, which demonstrates high potency (IC_50_ = 0.0065 μM) and approximately 1400-fold selectivity for NHE1 over NHE2. This compound also exhibits a favorable pharmacokinetic profile, including an oral bioavailability of 52% and a plasma half-life of 1.5 h [77]. Table 3 provides a summary on the current NHE1 inhibitors, their selectivity, clinical development stage, pharmacokinetics and major limitations. 

## 5. Translational Considerations for NHE1 Inhibition in Cancer

Prior clinical experience with NHE1 inhibitors provides an important foundation for translational exploration. As discussed above, multiple NHE1 inhibitors, including amiloride, cariporide, and eniporide, have been administered to thousands of patients in advanced phase II and phase III clinical trials for cardiovascular indications [78,79,80,83,85]. These studies established the feasibility of systemic NHE1 inhibition in humans and provided well-characterized safety and pharmacokinetic data. However, these studies were conducted outside the oncology context, and their outcomes cannot be directly extrapolated to cancer therapy.

In the oncology setting, effective NHE1 inhibition may require higher doses, prolonged exposure, or sustained target engagement to elicit downstream effects, including metabolic reprogramming. Transient or partial inhibition may be insufficient to induce metabolically actionable phenotypes, particularly given the complexity of the tumor microenvironment compared with cardiovascular targets [86]. These requirements raise important challenges related to dosing, drug delivery, and on-target toxicity. This distinction is particularly important given that many NHE1-dependent phenotypes in cancer appear to require sustained metabolic perturbation rather than acute signaling inhibition.

Such challenges are exemplified by amiloride, which is well tolerated at clinically approved doses but requires substantially higher exposures to achieve anticancer effects in preclinical models. These doses have been associated with adverse effects in vivo, including hyperkalaemia and cardiac arrhythmias resulting from altered renal potassium handling [69,87]. These observations highlight the relatively narrow therapeutic window of amiloride in cancer applications and underscore that efficacy, dosing, and tolerability in oncology cannot be assumed to directly translate from prior cardiovascular studies.

More selective NHE1 inhibitors may partially mitigate these limitations. Amiloride is a non-specific NHE1 inhibitor that also targets epithelial sodium channels in the kidney, contributing to its potassium-sparing effects and associated toxicities [69]. In contrast, HMA exhibits a 524-fold higher inhibitory potency toward NHE1 and more than an 80-fold reduction in urinary Na^+^/K^+^ ratios [69,88,89], making it an attractive compound for mechanistic studies of NHE1 function in cancer. However, HMA’s poor oral bioavailability and short plasma half-life currently limit its translational potential, emphasizing the need for next-generation NHE1 inhibitors that combine high potency with favorable pharmacokinetic profiles [33,73,74]. Beyond classical enzymatic inhibition of NHE1, targeted protein degradation strategies, such as lysosome-targeting chimeras (LYTACs), which direct membrane proteins to the lysosomal degradation pathway, may offer an alternative therapeutic approach [90]. The elevated expression of NHE1 in tumor tissues, as described earlier, may provide a theoretical therapeutic window that selectively stresses or inhibits cancer cells while sparing normal tissues. Advances can be made to current inhibitors to enhance their cancer specificity. For example, existing NHE1 inhibitors could be encapsulated within nanoparticles [91] or developed into tumor-specific antibody–drug conjugates [92]. Additionally, current NHE1 inhibitors could be engineered as prodrugs activated by the acidic tumor microenvironment [93]. Such strategies may enable tissue- or lesion-specific targeting of NHE1, thereby enhancing therapeutic precision while minimizing off-target effects.

Beyond pharmacological optimization, there is a growing need for metabolically informed preclinical studies that directly link NHE1 inhibition to defined metabolic vulnerabilities in cancer cells, such as cancer cell survival, stress responses, and treatment sensitivity [9,23,49]. While existing studies establish associations between NHE1 and specific metabolic endpoints, quantitative links between NHE1 inhibition and defined metabolic phenotypes remain limited. Specifically, determining the threshold, duration, and cellular context of NHE1 inhibition required to produce therapeutically meaningful metabolic effects represents an important and underexplored area of research. Given the consistent effects of NHE1 modulation on mitochondrial structure and function, mitochondrial remodeling represents a logical starting point for such systemic investigations.

These studies should be carried out using appropriate preclinical models, including cancer cell lines [94], organoids [95], animal models [96,97] and patient-derived xenografts (PDXs) [98] and may incorporate rational combination strategies with existing chemotherapeutic or immunotherapeutic regimens to investigate potential synergy. Given the broad involvement of NHE1 across a wide range of cancers (Table 1), comparative studies across malignancies may further clarify contexts in which NHE1-targeted strategies are most likely to be effective.

Alternatively, beyond tumor-wide approaches, translational efforts could be directed toward tumor types that are more likely to benefit from NHE1 inhibition. A strong starting point may be gliomas. Clinically, patients with high gene expression of *SLC9A1* exhibit significantly shorter overall survival compared to those with low expression levels. In addition, elevated *SLC9A1* expression correlates with higher tumor grade [7]. From a metabolic perspective, gliomas are highly dependent on mitochondrial metabolism [99]. Importantly, NHE1 perturbation in glioma models and the associated microglia resulted in impaired mitochondrial function and energy production [17,54]. Accordingly, gliomas may represent an attractive setting to deeply interrogate the therapeutic impact of NHE1 inhibition, particularly in combination with established chemotherapeutic agents or mitochondrial-targeting drugs.

In summary, while prior clinical experience establishes the feasibility of NHE1 inhibition in humans, challenges related to dosing, specificity, metabolic engagement, and combination strategies complicate their translation to oncology applications. Addressing these challenges will require rigorous, metabolically informed preclinical testing and the development of improved NHE1 inhibitors before clinical translation in cancer can be achieved.

## 6. Conclusions

NHE1 has emerged as a common regulator across multiple tumor types, affecting cell cycle progression, drug sensitivity, and cell death pathways. Although these phenotypes have been documented across diverse malignancies, the mechanistic basis by which NHE1 drives tumor progression remains unclear. Increasing evidence reviewed here supports altered cellular metabolism as a unifying theme underlying NHE1-dependent oncogenic phenotypes. In particular, NHE1 activity has been linked to dysregulated mitochondrial function and morphology, perturbations in lysosomal homeostasis, and altered redox balance, collectively shaping cancer cell stress responses and therapeutic vulnerability.

Advancing this field will require systematic and mechanistically informed investigation of metabolic consequences following NHE1 perturbation using appropriate preclinical models. In parallel, further work is needed to define the signaling pathways that couple NHE1 activity to metabolic remodeling and downstream tumor-promoting behaviors. Clarifying these mechanisms could reveal a previously underappreciated ion exchanger-metabolism-oncogenic signaling axis with broad relevance across cancer types. Importantly, the prior clinical evaluation of multiple NHE1 inhibitors provides a strong translational foundation, positioning this pathway as a promising target for future metabolically informed therapeutic strategies.

## Figures and Tables

**Table 1:** NHE1 is implicated across a wide range of solid and hematologic malignancies.

Cancer Type	Model Used	How NHE1 Was Investigated (Inhibitor/KD/KO, etc.)	Signaling Pathway Altered/Mechanism	Effect on Disease Outcome	Ref.
Breast cancer	Non-invasive human breast cancer cell line MCF-7 and highly invasive human breast cancer cell line MDA-MB-231.	Inhibition of NHE1 activity with cariporide	NHE1-mediated invasion and activity, and expression of membrane-type 1 matrix metalloproteinase (MT1-MMP)	Inhibition of NHE1 activity suppressed MDA-MB-231 cell invasion as well as activity and expression of MT1-MMP.	[25]
Colon cancer	Human colon adenocarcinoma cells LS174. Tumor xenografts with LS174 cell line in mice.	Genomic knockout of NHE1	Tumor cell proliferation. Tumor growth in mice.	NHE1 knockout significantly reduced tumor cell proliferation both in normoxia and hypoxia. Tumor xenografts revealed substantial reductions in tumor growth with NHE1 knockout.	[26]
Gastric cancer	GES-1, SGC-7901, and MKN-45 cells	Knockdown of NHE1 in MKN-45 and SGC-7901 cells. Pharmacological inhibition with 5-N-ethyl-N-isopropylamiloride (EIPA) in MKN-45 and SGC-7901 cells.	G1/S and G2/M cell cycle phase transition and proliferation	In these cells, NHE1 inhibition significantly suppressed gastric cancer cell proliferation, migration and invasion. Gastric cancer cell growth was suppressed in mice when SGC-7901 cancer cells were injected into nude mice with EIPA injection.	[9]
Ovarian cancer	Tissue samples from patients with epithelial ovarian cancer. OVCAR-3, 3AO, SKOV3 and A2780 distinct tumor-derived human ovarian cancer cell lines.	5-year follow-up study on clinical prognosis of EOC. Expression of NHE1 in cell lines.	Not applicable	Increased expression of NHE1 was associated with advanced stage and high-grade carcinoma.	[6]
Cervical cancer	Normal human cervical epithelial cells, cervical cancer SiHa and CaSki cell lines	Epidermal growth factor (EGF) supplementation	Epidermal growth factor upregulation of NHE1 abundance	Upregulation of NHE1 by EGF increased cervical cancer cell invasiveness	[27]
Esophageal cancer	Human esophageal squamous cell carcinoma (ESCC) cell lines TE2 and TE5	Knockdown with NHE1 siRNA in TE2 and TE5 cells. NHE1 overexpression in TE2 and TE5 cells.	Not applicable	Knockdown of NHE1 in ESCC cells promoted cell growth, invasion, and migration. NHE1 overexpression in TE2 cells and TE5 cells inhibited cell growth and induced apoptosis.	[8]
Pancreatic cancer	Human pancreatic cancer cell lines: PANC-1, BxPC-3, MiaPaCa-2, and CAPAN-2	Epidermal growth factor (EGF) supplementation, NHE1 inhibition with cariporide	Interaction of NHE1 with epidermal growth factor receptor (EGFR)	Inhibition of NHE1 reduced three-dimensional growth and invasion independently of pancreatic ductal adenocarcinoma subtype and synergistically sensitized the effects to low doses of erlotinib	[28]
Brain cancer	Chinese Glioma Genome Atlas (CGGA) dataset containing transcriptome sequencing data and Cancer Genome Atlas (TCGA) containing mRNAseq data. Mouse SB28 and GL26 intracranial syngeneic glioma models.	mRNA expression was examined in the datasets. NHE1 inhibition with HOE642 (cariporide) in mice.	Activating CD8 T-cell accumulation, increasing expression of interferon-gamma	Higher NHE1 mRNA levels were found in higher grade gliomas and worsened survival probabilities were correlated with the elevated NHE1 mRNA levels in gliomas. Inhibition of NHE1 reduced glioma volume, invasion, and prolonged overall survival in mouse glioma models. Animals were sensitized to anti-PD-1 therapy.	[7]
Lung cancer	Drug-resistant human small cell lung cancer H446/CDDP cells	Recombinant NHE1 antisense gene was transfected into cells	Not applicable	NHE1 antisense gene induced cells to become acidified and apoptotic	[29]
Acute myeloid leukemia	MV4-11, MOLM13, THP-1, Kasumi-1, KG-1alpha, 293T cell lines. NOD/SCID female mice injected with MV4-11 cells.	Knockdown of NHE1 in MV4-11 cells. Pharmacological inhibition of NHE1 in MV4-11, MOLM13, THP-1, Kasumi-1 and KG-1alpha cells with HMA, venetoclax, or combination of HMA and venetoclax. MV4-11-luc+ cells xenografted in mice and treated with HMA, venetoclax or combination of HMA and venetoclax.	Cell cycle arrest, proliferation and viability.	Knockdown of NHE1 in MV4-11 cells led to a significant decrease in cell viability. HMA inhibition dose-dependently arrested the cell cycle in MV4-11 and MOLM13 cells. HMA+ venetoclax dramatically decreased the proportion of proliferating MV4-11 and MOLM13 cells. Cell viability was decreased in Kasumi-1, KG-1alpha and THP-1 cells treated with both HMA and venetoclax.	[30]
T-cell acute lymphoblastic leukemia	Human T-ALL MOLT4 cell line	NHE1 siRNA transfection. Pharmacological inhibition of NHE1 with cariporide. CCL25 stimulation on expression of NHE1 in MOLT4 cells.	Expression of NHE1, migration of MOLT4 cells.	NHE1 knockdown increased the sensitivity of MOLT4 cells to doxorubicin. CCL25 stimulation induced the increased expression of NHE1. NHE1 silencing decreased CCL25-induced migration in MOLT4 cells.	[31]
Chronic myeloid leukemia	K562 CML cell line	NHE1 shRNA and pharmacological inhibition of NHE1 with cariporide.	ERK1/2 mediated cellular differentiation	Inhibiting NHE1 with CIAPIN1 promoted differentiation of K562 cells	[32]
Multiple myeloma	RPMI-8226, U266, MM.1S and ARH-77 cell lines	NHE1 knockout and pharmacological inhibition of NHE1 with HMA.	Transcription Factor E3 mediated lysosome biogenesis.	Higher NHE1 gene expression was associated with shorter survival in multiple myeloma patients. Knockout of NHE1 was associated with increased lysosomal membrane permeability in RPMI-8226/NHE1 KO cells HMA reduced the pH, inhibited proliferation and induced apoptosis of multiple myeloma cell lines HMA induced lysosomal rupture in multiple myeloma cell lines	[33]

CGGA: Chinese Glioma Genome Atlas, CML: Chronic myeloid leukemia, EGF: Epidermal growth factor, EGFR: Epidermal growth factor receptor, EIPA: 5-N-ethyl-N-isopropylamiloride, EOC: Epithelial ovarian cancer, ERK1/2: Extracellular signal-regulated kinases 1 and 2, ESCC: Esophageal squamous cell carcinoma, HMA: 5-(N-hexamethylene)amiloride, KD: Knockdown, KO: Knockout, MT1-MMP: Membrane-type 1 matrix metalloproteinase, NHE1: Na^+^/H^+^ exchanger 1 (SLC9A1), NOD/SCID: Non-obese diabetic/severe combined immunodeficiency, PD-1: Programmed cell death protein 1, shRNA: Short hairpin RNA, siRNA: Small interfering RNA, T-ALL: T-cell acute lymphoblastic leukemia, TCGA: The Cancer Genome Atlas.

**Table 2:** NHE1 modulation reveals conserved metabolic vulnerabilities across several cancer types.

Metabolic Process/Organelle Affected	Cancer Type	Model Used	NHE1 Modulation (Inhibitor/KD/KO, etc.)	Key Findings	Ref.
OXPHOS	Pancreatic cancer	BxPC3	Pharmacological inhibition–EIPA	Treatment resulted in decreased basal and maximal respiratory capacity compared to normal untransformed cells	[52]
	Glioblastoma	GAMs in mouse glioma model	Pharmacological inhibition-cariporide	Combination treatment of TMZ and HOE642 upregulated the OXPHOS pathway genes	[17]
Mitochondria	Breast cancer	MCF7 cells	Pharmacological inhibition–HMA	Treatment resulted in mitochondrial swelling, fragmentation and perinuclear accumulation	[51]
	Pancreatic cancer	BxPC3	Pharmacological inhibition-EIPA	Altered morphology and the formation of elongated tubular network	[52]
	Breast cancer	MDA-MBA-157	Pharmacological inhibition-EIPA	Altered morphology and the formation of elongated tubular network	[52]
	Glioblastoma	GAMs in mouse glioma model	Pharmacological inhibition-cariporide	Combination treatment of TMZ and HOE642 increased mitochondrial mass	[17]
	Colon cancer	HCT-116 cells	Pharmacological inhibition–HMA	Rearrangement in mitochondrial structure, increased perinuclear localization and mitochondrial membrane depolarization	[18]
	Mesothelioma	H-2452AcT and H-2452 cells	Pharmacological inhibition–cariporide	ROS accumulation and loss of mitochondrial membrane potential	[53]
	Glioblastoma	U87Mg cells	Pharmacological inhibition-UCD38B	Reduction in mitochondrial membrane potential	[54]
Lysosome	Breast cancer	MCF7, MDA-MB-231 and Met-1 cells	Pharmacological inhibition–HMA	Inhibition of ROS and lysosomal protease rescued the cells from HMA-induced cytotoxicity	[51]
Glycolysis	Glioblastoma	GAMs in mouse glioma model	Pharmacological inhibition–cariporide	Increased glucose uptake and mitochondrial mass; decreased aerobic glycolysis (TMZ + HOE642)	[17]
	Acute myeloid leukemia	NOMO-1 and MV4-11 cell lines	Pharmacological inhibition–HMA	Decreased glucose uptake	[16]

OXPHOS: Oxidative phosphorylation, HOE642: cariporide, GAMs: Glioma-associated microglia/macrophages, TMZ: Temozolomide, ROS: Reactive oxygen species, EIPA: 5-N-ethyl-N-isopropylamiloride, HMA: 5-(N-hexamethylene)amiloride.

**Table 3:** Summary of selected NHE1 pharmacological inhibitors.

NHE1 Inhibitor	Inhibitory Potency to NHE1 (μM)	Selectivity	Clinical Development Stage	Pharmacokinetics	Major Limitations	Ref.
Amiloride	1–1.6	Inhibits ENaCs, Na^+^/Ca^2+^ exchanger and uPA in addition to NHE1	FDA Approved (cardiovascular indications)	F = 50% tmax: 2–3 h t1/2: 6–9 h	Limited NHE1 specificity; off-target inhibition of ENaCs, Na^+^/Ca^2+^ exchanger, and uPA. Anticancer effects require high doses, increasing the risk of adverse effects such as electrolyte imbalance	[62,67,68,71,72]
HMA	0.013	Reduced inhibition of ENaCs and the Na^+^/Ca^2+^ exchanger relative to amiloride	Pre-clinical	F = 4.5% t1/2: 36 min	Poor oral bioavailability and short plasma half-life	[62,73]
EIPA	0.01–0.02	Reduced inhibition of ENaCs and the Na^+^/Ca^2+^ exchanger relative to amiloride	Pre-clinical	t1/2: 31 min Administered intraperitoneally in preclinical models	Short plasma half-life and limited in vivo pharmacokinetic data	[62,74]
Cariporide	0.03–3.4 (assay-dependent)	Minimal reported inhibition of Na^+^ channels or the Na^+^/Ca^2+^ exchanger	Phase II (cardioprotection)	t1/2: 3.5 h (human) t1/2: 40–80 min (rat) Parenteral administration	Increased mortality and cerebrovascular events observed at higher doses in the EXPEDITION trial	[62,78,79,80,81]
Eniporide	0.005–0.38	Minimal reported inhibition of Na^+^ channels or the Na^+^/Ca^2+^ exchanger	Phase III (cardioprotection)	t1/2: 2 h (human) Parenteral administration	Limited cancer-specific preclinical data	[62,82,83,84]
Compound 9t	0.0065	Limited published data on off-target effects currently available	Pre-clinical	F = 52% t1/2: 90 min	Limited data on cancer-specific testing	[77]

NHE1: Sodium–hydrogen exchanger 1, ENaCs: epithelial sodium channels, Na^+^/Ca^2+^ exchanger: sodium–calcium exchanger, uPA: urokinase-type plasminogen activator, HMA: 5-(N-hexamethylene)amiloride, EIPA: 5-(N-ethyl-N-isopropyl)amiloride, F: oral bioavailability, tmax: time to peak plasma concentration, t1/2: elimination half-life, IP: intraperitoneal, FDA: U.S. Food and Drug Administration.

## Data Availability

Not applicable.

## Abbreviations

The following abbreviations are used in this manuscript:

AML	Acute myeloid leukemia
CGGA	Chinese Glioma Genome Atlas
CML	Chronic myeloid leukemia
Cr	Creatine
ECAR	Extracellular acidification rate
EGF	Epidermal growth factor
EGFR	Epidermal growth factor receptor
EMT	Epithelial–mesenchymal transition
ENaCs	Epithelial sodium channels
EIPA	5-N-ethyl-N-isopropylamiloride
EOC	Epithelial ovarian cancer
ERK1/2	Extracellular signal–regulated kinases 1 and 2
ESCC	Esophageal squamous cell carcinoma
FDA	U.S. Food and Drug Administration
GAMs	Glioma-associated microglia
HMA	5-(N-hexamethylene)amiloride
IFN-γ	Interferon gamma
JNK	c-Jun N-terminal kinase
KD	Knockdown
KO	Knockout
LAMC2	Laminin subunit gamma 2
MAPK	Mitogen-activated protein kinase
MM	Multiple myeloma
MT1-MMP	Membrane-type 1 matrix metalloproteinase
NHE	Na^+^/H^+^ exchanger
NHE1	Na^+^/H^+^ exchanger 1 (SLC9A1)
NHERF1	Na^+^/H^+^ exchanger regulatory factor 1
NOD/SCID	Non-obese diabetic/severe combined immunodeficiency
OCR	Oxygen consumption rate
OXPHOS	Oxidative phosphorylation
PCr	Phosphocreatine
PD-1	Programmed cell death protein 1
PD-L1	Programmed death-ligand 1
PDXs	Patient-derived xenografts
ROS	Reactive oxygen species
shRNA	Short hairpin RNA
siRNA	Small interfering RNA
T-ALL	T-cell acute lymphoblastic leukemia
TCGA	The Cancer Genome Atlas
TCA	Tricarboxylic acid (cycle)
TMZ	Temozolomide
uPA	Urokinase-type plasminogen activator
